# 2817. Outcomes of Timely vs. Delayed Meropenem/Vaborbactam Treatment of Infections with CRE among Adults Hospitalized in the US, 2019-2021

**DOI:** 10.1093/ofid/ofad500.2428

**Published:** 2023-11-27

**Authors:** Marya D Zilberberg, Brian H Nathanson, Mark Redell, Kate Sulham, Andrew Shorr

**Affiliations:** EviMed Research Group, LLC, Goshen, Massachusetts; OptiStatim LLC, Longmeadow, Massachusetts; Melinta Therapeutics, Morristown, NJ; Melinta Therapeutics, Morristown, NJ; Medstar Washington Hospital Center, Not Applicable

## Abstract

**Background:**

Carbapenem-resistant Enterobacterales (CRE) represent an urgent threat. Mortality rates associated with this pathogen approach 80%. Timely treatment of serious infections, even with highly resistant organisms, among hospitalized patients improves the chances of survival. We explored whether meropenem/vaborbactam (MEV) conforms to this observation.

**Methods:**

We conducted a multicenter retrospective cohort study in ∼300 hospitals reporting microbiology data within the Premier Healthcare Database, 2019-2021, to explore the effect of timely (MEV-T) vs. delayed (MEV-D) CRE treatment. We included all adult hospitalized patients who either upon admission or during hospitalization had sepsis, a UTI, a cIAI, or pneumonia, and had at least one corresponding culture positive for CRE. Treatment was MEV-T if instituted within 2 days of obtaining the index culture. All other was considered MEV-D.

**Results:**

Among 1,513 patients with CRE, 87 (5.8%) received MEV. Among those treated with MEV, 29 (33.3%) were treated in a timely manner. At baseline, MEV-T patients were directionally sicker than those in the MEV-D group, as evidenced by a higher prevalence of DNR on admission and a higher mean Charlson Comorbidity score (Table). Similarly, MEV-T patients were slightly more likely to require admission to an intensive care unit and mechanical ventilation. Although not statistically different, hospital mortality trended lower with MEV-T relative to MEV-D. Rates of incident *C. difficile* infection (CDI) and acute kidney injury (AKI) were lower with MEV-T (Table). Mean post-infection hospital length of stay (14.0+/-10.3 vs. 25.4+/-25.0 days, P=0.021) and hospital costs ($85,776+/-$81,454 vs. $149,506+/-163,748, P=0.052) were lower with timely MEV therapy.

Table

**Figure:**
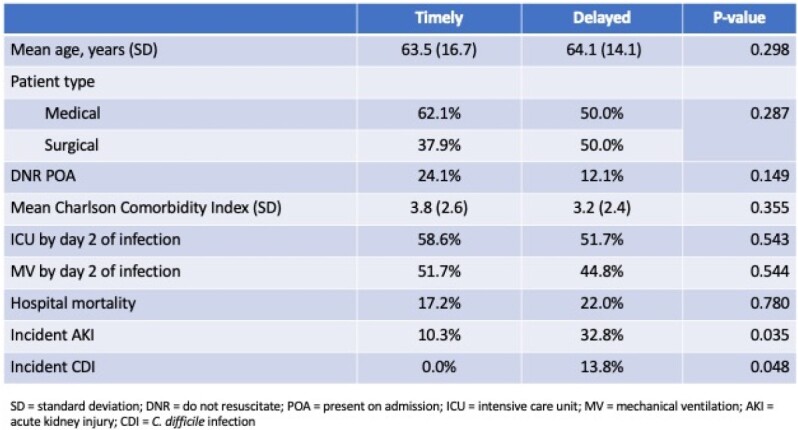

**Conclusion:**

Despite being more severely ill, the group treated with timely initiation of MEV for CRE resulted in a significantly lower incidence of CDI and AKI. The post-infection LOS and hospital costs were also lower in the MEV-T group. Hospital mortality trended lower in the MEV-T group. Despite the small sample size, these differences suggest that timely administration of MEV may improve select significant clinical outcomes.

**Disclosures:**

**Marya D. Zilberberg, MD, MPH**, Melinta Therapeutics: Grant/Research Support|Merck: Grant/Research Support|scPharmaceuticals: Advisor/Consultant|scPharmaceuticals: Grant/Research Support **Brian H. Nathanson, Ph.D.**, Merck & Co., Inc: Advisor/Consultant **Mark Redell, PharmD**, Melinta Therapeutics: Full-time employee|Melinta Therapeutics: Full-time employee|Melinta Therapeutics: Stocks/Bonds|Melinta Therapeutics: Stocks/Bonds **Kate Sulham, MPH**, Melinta Therapeutics: Advisor/Consultant

